# Ophthalmology

**Published:** 1896-08

**Authors:** Alvin A. Hubbell


### OPHTHALMOLOGY.

CONDUCTED BY ALVIN A. HUBBELL, M. D.

### TREATMENT OF CONVERGENT STRABISMUS.

PRIESTLY SMITH, of Birmingham, in the Ingleby Lectures for 1896, (*British Medical Journal*, June 27, 1896,) gives his views of the treatment of strabismus as follows :

*Will glasses cure the squint ?* If there be a considerable hypermetropia in the straight eye, glasses will probably diminish the deviation whatever be the condition of the deviating eye ; but if this latter fail to right itself when the fellow-eye is covered, they will not effect a real cure. The more promising cases are those in which the squint alternates between the two eyes, the most promising those in which it appears and disappears periodically. In a case of this kind, if we find that atropine vanishes it entirely for the time being, we may be almost sure that glasses will remove it permanently. But the glasses must be correct, neutralising astigmatism as well as hypermetropia in both eyes ; they must " fit " so that the child looks through them and not over them, and they must be worn constantly from morning till night. Failure frequently depends on lack of perseverance. The parents want a speedy cure ; not getting it, they allow the child to lay aside the glasses after a few weeks or months or fail to renew them when he breaks them. Sometimes they complain, illogically, that " the glasses are no good, for the squint returns the moment they are taken off." In reality this is the strongest proof of their value. But we must not expect too much from glasses ; they can lessen the excess of convergence and they can sharpen the retinal pictures, but they cannot create the visual perceptions and motor impulses which are essential to the acquirement of binocular vision.

*What else can be done ?* The squinting eye, or rather the perceptions and impulses which ought to control its movements, can, to some extent at least, be educated. The process will be difficult or impossible according to the duration of the previous neglect. The patient must be taught, first, to perceive the impressions belonging to the squinting eye; then to perceive them simultaneously with those of the fellow-eye—that is, to see double; next, to make the effort which is necessary to unite the double images; and lastly, to form those judgments of distance and perspective which perfect binocular vision gives. Cures absolutely complete in this last respect, as proved by Hering's drop test, are rare but not unknown. For this process of teaching several means are at our disposal. We may cause the patient to use the squinting eye exclusively by covering the other; we may induce him to give it preference, when both are open, by partially disabling the other with atropine; we may teach him to notice both pictures simultaneously and ultimately to blend them by means of persistent practice with the stereoscope, differentiating them at first by colored glasses and approximating them by prisms; we may facilitate the process at any stage by bringing the eye into better position by means of tenotomy.

*When is it best to operate?* No operation should be done until the nature of the squint has been thoroughly investigated and until efforts have been made to minimise the deviation and to sharpen the visual perceptions by glasses and by regulated practice. When these measures are inapplicable, or have ceased to give progressive benefit, operation is in place and is hardly contra-indicated by age in either direction; but the younger the child the more important it is to do too little rather than too much. Full correction of a squint by tenotomy in very early life may give over-correction later on. Partial correction by the first tenotomy, an interval with regulated exercise, and then a similar tenotomy on the fellow-eye in case of need are better practice. Confirmed strabismus often diminishes in degree as time goes on; hence the too common idea that time alone will often cure a squint and the advice not seldom given to "wait and see." For the squinting eye this too often means waiting and not seeing. Time alone may lessen the disfigurement but will not remove the disability. Binocular vision is not recovered in this way.

---

ON THE PREPARATION OF MACROSCOPICAL EYE SPECIMENS.

Dr. William Campbell Posey, of Philadelphia, (*Annals of Ophthalmology and Otology*, January, 1896,) in preparing macroscopical eye specimens, uses a simple glass jar made by the Fox Optical Co. and capped with a plano-convex lens. A small disc of opaque glass is used to seal the jar and forms an excellent background for the specimen.

The preparation of the eye for mounting is briefly as follows: The entire globe is hardened in Muller's fluid for at least three weeks. It is then placed in a freezing mixture of salt and ice, care being taken to envelop the eye in oiled silk to prevent any of the fluid from the freezing mixture coming in contact with it. After the globe is thoroughly hardened it is divided by a bold stroke of a knife,—that form of knife used in sectioning the brain being the best adapted for this purpose. One-half of the eye is reserved for microscopical examination, whilst that which is to be mounted macroscopically is placed in water to thaw out and to rid it of the excess of Muller's fluid. To remove the stain caused by the fluid more effectually the section of eye is placed in a 5 per cent. solution of chloral hydrate, care being taken to change the solution daily until the section is no longer discolored. As the globe would shrink were it placed in the mounting fluid at this stage, it is first subjected to solutions of glycerine and water of different strengths. The eye is then ready for mounting.

The preparation of the medium used for this purpose is the only difficult stage in the entire process. It is quite a painstaking task to obtain a clear solution, and one frequently consumes three or four hours in the process, to be rewarded by a product which is too cloudy for use. The solution is best prepared as follows: One ounce (about twelve to fourteen strips) of the best French gelatin (Coignet & Co., Paris,) is cut into small bits by a pair of scissors; eight ounces of water is then added to the gelatin and the mass set aside in a beaker to swell. The whites of two eggs are then taken, and to better clear the mixture later on, the shells of the eggs are cut into fine pieces and thoroughly mixed with the albumen. The gelatin is now melted with a gentle heat, a few drops of carbolic acid being added as an antiseptic. As soon as it begins to boil, the albumen is added and the whole mass stirred with a glass rod. As the mixture is very rapidly scorched, constant attention must be given it until the fluid is quite clear below and a thick scum has gathered upon the top. The mixture is now filtered through flannel, or preferably through a steam filter, and the product, which should be quite clear, is collected in a jar which has been made perfectly clean. Eight ounces of glycerin is then added. After the glycerin and jelly have been thoroughly mixed one of the glass cells is two-thirds filled with the solution and the section of eye floated upon it. The globe is then seized at the equator with a pair of forceps and gently rolled over in such a way that no air bubbles are permitted to get into its concavity. Should any bubbles, however, get in they can be readily removed by sucking them out through a glass tube. A hand-mirror is now placed under the specimen and a good view is obtained of the position of the eye and of any air bubbles which may have escaped detection from above. The globe is then kept from rising in the fluid by placing over it a bit of cardboard perforated by a pin.

The jar should never be entirely filled with the solution, for it is always better to leave an air space between it and the opaque disc, which is cemented on after the jelly has thoroughly set.

---

### LIGHT—THE OINTMENT OF THE YELLOW OXIDE OF MERCURY—AND THE OINTMENT POTS IN COMMON USE.

Dr. Holth, of Norway, (*Archives of Ophthalmology*, October, 1895,) calls attention to the fact that the yellow oxide of mercury ointment decomposes very readily. He says:

It is a familiar fact that this ointment decomposes quite readily ; a change takes place after a short period, sometimes at the end of a few days. As a result, there is grayish discoloration, which, becoming darker, finally changes to black. Altered in this manner, the ointment not only suffers in appearance, but, though possibly harmless, it is less potent and may even be entirely useless. If its use be limited to a few days, or if it be freshly prepared every day for use in clinics, there will be practically no decomposition ; but as ordinarily prescribed to be applied at home for a considerable period of time this change is an objectionable feature.

The writer conducted a series of experiments for the purpose of ascertaining the cause of this decomposition. His results agree with the statements found in text-books of chemistry, that the red and yellow oxides of mercury are reduced through the influence of light. They justify the following conclusions:

1. Decomposition of the ointment of the yellow oxide of mercury, showing itself in grayish discoloration, is not due to chemical changes produced in the ointment base, but is dependent entirely upon the reducing effect of light passing through the more or less transparent walls of the ointment pots in ordinary use. Violet light probably exerts the most effect in this direction.

2. This decomposition is not influenced by temperatures between 0° and 30° C.

3. It is not caused by access of air.

4. Any ointment pot may be used if kept in a dark place.

5. The use of absolute opaque pots provided with similar covers is the most practical way of avoiding decomposition.

———

### EPHEDRENE HYDROCHLORAT AS A MYDRIATIC.

Dr. George F. Suker, of Toledo, Ohio, (*Annals of Ophthalmology and Otology*, January, 1896,) writes that ephedrene is an alkaloid from the leaves of ephedra vulgaris or ephedra distachya, and not to be confounded with ephedren, which latter is an amorphous compound obtained in connection with glucose during the decomposition of the glucosoide present in ephedra antisyphilitica. This ephedrene is considered the active principle of the plant. Kindly note the difference in the spelling of the two—ephedren and ephedrene.

The alkaloid ephedrene is not amorphous, but a colorless stable crystal, melting at 410° F. (210° C.), soluble in four parts of water, readily so in alcohol, practically insoluble in ether. The hydrochlorid, in which form the base is chiefly used, appears in colorless needles, very readily soluble in water.

It was Mr. Kinnosuke Miura, of Tokio, who first found the aqueous solution of ephedrene. The clinical researches of Kinnosuke Miura first demonstrated the mydriatic properties of ephedrene hydrochlorat when applied externally in frogs and mammals, as well as evincing its lethal effect when a sufficient quantity was taken internally.

It causes death by the arrest of the heart's action and respiration. He reported a series of eighteen cases of the use of a 10 per cent. solution, carefully noticing its mydriatic properties. In each and every case he noticed a uniformity of action as well as a good reliability. The suspension of accommodation was not perceptible. In each case one or two drops of a 10 per cent. solution were instilled into the conjunctival sac at a few moments' interval, and a complete mydriasis was obtained in ten to twenty minutes.

Of late, Dr. Groenouew and Dr. Geppert have made extended clinical experiments, and these confirm fully those of Miura. However, they use the ephedrene in connection with the homatropin. They say that the addition of homatropin greatly increases the intensity of the ephedrene, yet not influencing its transitory effects. The following is the formula which they employ: Ephedene hydrochloride, 1.0; homatropin hydrobromide, 0.01; aqua distilled, 10.0.

---

### CONTRIBUTIONS TO THE PATHOLOGICAL ANATOMY AND BACTERIOLOGY OF SUPPURATIVE KERATITIS IN HUMAN BEINGS.

UNTHOFF and AXENFELD (*Archiv. f. Ophth., Abth.* I., 1896; *Annals of Ophthalmology and Otology*, April, 1896,) contribute an article of 130 pages, going extensively into the pathology and describing twenty-four cases. These include superficial and perforating ulcers, hypopyon keratitis, keratomalacia, panophthalmitis, and the like, of local origin or appearing in the course of general diseases from different forms of infection. There were 35 cases of typical ulcus corneæ serpens, 10 cases of hypopyon, 1 case of keratomycosis aspergillina, 2 cases of beginning panophthalmitis with corneal abscess. In these there were found the Frankel-Weichselbaum diplococcus (pneumococcus) in 26 cases, of which 4 cases were typical ulcus corneæ serpens and 2 cases beginning panophthalmitis; pneumococci at the same time with other microorganisms in 7 cases; 5 of these were typical ulcus serpens; no pneumococci but other microörganisms in 13 cases; 4 cases were typical ulcus serpens; negative bacteriological findings in 4 cases, 2 of these ulcus serpens. There was 1 case of keratomycosis aspergillina, arising from dirt being thrown in the eye, causing a typical hypopyon keratitis. The eye was not enucleated although bacteriologic examination of the pus was made. Experimental inoculation of animal corneæ was done in all cases and results noted. The literature is extensively quoted.

---

### FORMIC ALDEHYDE IN OPHTHALMIC PRACTICE.

JAMES MACKENZIE DAVIDSON, M. B., C. M., Aberdeen (*British Med. Journal*, January 18, 1896).

The writer cites a number of cases of corneal abrasions and ulcers, in two instances with hypopyon, which were treated with a solution of formalin 1 to 2,000 or 3,000 with most favorable results.

The results which I have obtained with this substance in the treatment of some diseases of the eye have been so notable that I am induced to publish this short article upon its use. The preparation I am using is Shering's formalin (which consists of 40 per cent. formic aldehyde in water, forming a stable solution, if kept in a well-stoppered bottle). One part of formalin in 2,000 or 3,000 of water is the strength which I find most serviceable. When I tried it first in hypopyon ulcers it was dropped into the affected eye three or four times daily and it seemed to be of very little use, but on applying it freely every hour I have never seen anything act so effectually in these cases.

---

### ETIOLOGY OF PTERYGIUM.

SCHULEK (*Ungar. Beitr. z. Augenheilk., I.*, 1895; *Annals of Ophthalmology and Otology*, April, 1896,) in two articles shows that pterygia arise from irritating causes, such as intense light or temperature and dust, and endeavors to show that the irritating qualities of the ultra violet rays of light (which exist in white light) are an important factor. Most foreign bodies, such as dust particles, remain longest upon the nasal side of the conjunctiva. Pterygia start from infolding of the conjunctiva upon small erosions at the corneal limbus. Continued irritation causes growth. Foreign bodies are frequently found at the point of the pterygium. Pinguecule frequently becomes transformed into pterygium. The ultra violet rays affect the cornea and conjunctiva by photochemical irritation.

---

EFFERVESCENT LITHIA TABLETS.—The varying and uncertain composition of natural lithia waters precludes an intelligent medical use. If the dose can even be approximated, it is altogether too small in the volume of water required to contain it to be available, practically. Not so, however, with the lithia tablet—the effervescent citrate of lithia tablet—as made by Messrs. John Wyeth & Brother, chemists, Philadelphia, in each of which there is an exactly definite proportion of the salt, an absolute uniformity of dose, with therapeutic result assured. These tablets, which have a widely extended popularity, and such unreserved endorsement of the medical profession, present evidences of superiority which explain the preference everywhere accorded them. The tablets dissolve with free, sparkling effervescence, affording a bright, clear solution and an agreeable drink.

